# Subanesthetic isoflurane abates ROS-activated MAPK/NF-κB signaling to repress ischemia-induced microglia inflammation and brain injury

**DOI:** 10.18632/aging.202349

**Published:** 2020-12-28

**Authors:** Zhiqiang Yao, Ningning Liu, Xiaoshan Zhu, Ling Wang, Yali Zhao, Qinqin Liu, Chunfang Gao, Juntang Li

**Affiliations:** 1 Department of Interventional Neuroradiology, The First Affiliated Hospital of Zhengzhou University, Zhengzhou 450052, Henan, China; 2 Department of Neurosurgery, Zhujiang Hospital, Southern Medical University, Guangzhou 510282, Guangdong, China; 3 Centre of Inflammation and Cancer Research, 150th Central Hospital of PLA, Luoyang 471031, Henan, China; 4 Department of Anesthesiology, 150th Central Hospital of PLA, Luoyang 471031, Henan, China; 5 Department of Immunology, The Fourth Military Medical University, Xi’an 710032, Shaanxi, China

**Keywords:** isoflurane, ischemia, inflammation, ROS, microglia, neuron

## Abstract

Isoflurane (ISO) elicits protective effects on ischemia-induced brain injury. We investigated whether sub-anesthetic (0.7%) ISO post-conditioning attenuates the inflammation and apoptosis in oxygen-glucose deprivation (OGD)-insulted co-cultures (microglia and neurons) *in vitro* and the brain injury of the middle cerebral arterial occlusion (MCAO) rat. We demonstrated that ISO augmented the viability of OGD-treated microglia and neurons. ISO reduced the expression and activation of COX2 and iNOS in OGD-challenged microglia. ISO repressed the production of tumor necrosis factor-α, interleukin (IL)-1β, IL-6, IL-8, and monocyte chemoattractant protein-1 in OGD-exposed microglia. ISO also decreased nucleosomal fragmentation and caspase-3 activity but increased mitochondrial membrane potential in OGD-stimulated microglia and neurons. Mechanistically, ISO suppressed OGD-induced microglial inflammation by blocking ROS-regulated p38 MAPK/NF-κB signaling pathway and hampered OGD-triggered microglial apoptosis in a ROS- or NO-dependent fashion. *In vivo* results with MCAO rats were partly consistent with the *in vitro* observation. These findings indicate that sub-anesthetic ISO post-conditioning abates the inflammation and apoptosis in OGD-stimulated rat microglia and the apoptosis of OGD-exposed neurons and the brain injuries of MCAO rats, suggesting it as a potentially effective therapeutic approach for ischemic brain damages.

## INTRODUCTION

Hypoxic-ischemic brain diseases are the leading causes of morbidity and mortality in humans. Dysregulation of inflammatory response and enhanced oxidative stress and apoptosis are observed in ischemic brain injuries [1]. The inflammatory factors are implicated in ischemia-related microglia and neuron death [2]. Thus, reducing inflammatory mediators is a potential strategy to treat ischemic brain damage.

Microglia are unique resident immune cells of the central nervous system (CNS) that defend against ischemia [3]. In physiological condition, resting microglia play a key role in the homeostasis within the CNS [4]. Upon activation, microglia secrete excessive pro-inflammatory factors including tumor necrosis factor α (TNF-α), interleukin (IL)-1β, and IL-6, and produce considerable reactive oxygen species (ROS) and nitric oxide (NO), which cause the death of microglia and neurons as well as the disruption of blood brain barrier [5–8]. ROS-mediated activation of mitogen-activated protein kinases (MAPKs) contributes to the production of inflammatory mediators in oxygen-glucose deprivation (OGD)-challenged BV-2 microglial cells [9]. OGD-activated microglia upregulate the expression of inflammatory factors via nuclear factor (NF)-κB, the transcription factor downstream of MAPK signaling [10, 11]. Reportedly, the inflammatory response is a highly regulated process in which the balance between cell survival and apoptosis is orchestrated to ultimately drive or resolve the inflammation [12]. Given the inflammation and apoptosis of activated microglia are central to ischemia-related brain pathology, limited information is available regarding the mechanisms by why reduce these pathological damages.

Isoflurane (ISO) is a commonly used inhaled anesthetic with anti-inflammatory, anti-oxidative, and anti-apoptotic properties [13, 14]. ISO reduces the production of pro-inflammatory cytokines and chemokines in zymosan-challenged neutrophils or Kupffer cells [15–18]. ISO alleviates OGD-induced BV-2 cell apoptosis [19]. ISO post-conditioning reduces brain infarct volume and atrophy and improves neurobehavioral outcomes in middle cerebral arterial occlusion (MCAO) rats by impeding inflammatory stress-caused microglial apoptosis [20, 21]. Yin et al. [22] reported that ISO post-conditioning-induced neuroprotection in ischemia-treated rat brains may depend on the reduction of inflammatory factors in microglia. In literature, ISO at clinical anesthetic dose (1.2%–2.5%) has adverse effects for critically ill patients who cannot tolerate its hemodynamic effects. ISO at less than 1% for sedation weakly interferes with hemodynamics, which is more beneficial for ill patients [23]. Previously, we demonstrated that sub-anesthetic dose (0.7%) of ISO post-conditioning ameliorates zymosan-induced lung injury in *vitro* and *in vivo* by inhibiting the inflammation and apoptosis [15, 16]. 0.7% ISO post-conditioning decreases zymosan-induced inflammation in the murine Kupffer cells by blocking ROS-activated p38 MAPK/ NF-κB signaling [17]. In this study, we investigated the beneficial effects and underlying mechanisms of sub-anesthetic ISO post-conditioning in reducing the inflammation and apoptosis in OGD-insulted microglia and the apoptosis of OGD-stimulated neurons as well as the brain injuries of MCAO rats.

## RESULTS

### Sub-anesthetic ISO post-conditioning reduces OGD-induced viability inhibition and apoptosis of microglial cells *in vitro*

**Figure 1:**
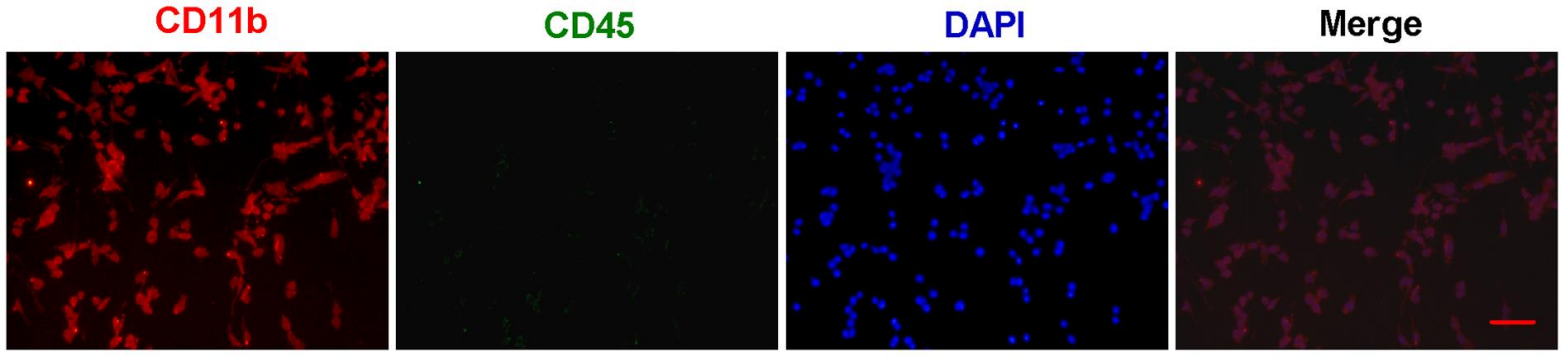
**The identification and purity of primary microglia.** Primary microglial cells were isolated from one-day-old SD rats and grown in DMEM/F12 medium. The identification and purity of microglial cells was determined by immunofluorescent staining with CD11b (Red). To distinguish microglia from macrophages, the double staining for CD11b and CD45 (Green) were performed. In merged images, all the CD11b-positive cells (Red) almost coincide with all the DAPI-positive cells (Blue). Scale bar: 20 μm. DAPI: 4',6-diamidino-2-phenylindole.

First, primary microglial cells were isolated from one-day-old SD rats and grown in DMEM/F12 medium. The identification and purity of microglial cells was determined by immunofluorescent staining with CD11b (Figure 1). To distinguish microglia from macrophages, we performed the double staining for CD11b and CD45 (Figure 1). And all the CD11b-positive cells almost coincided with all the DAPI-positive cells (Figure 1), suggesting that we obtain the very pure microglia.

**Figure 2:**
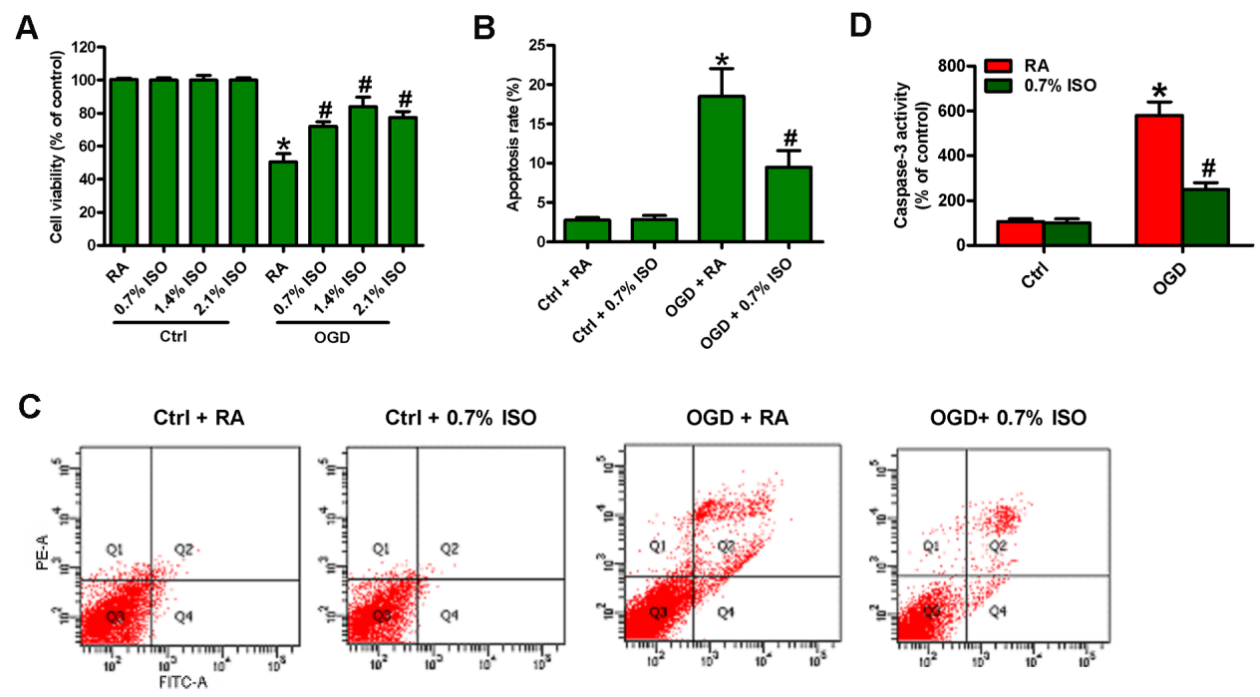
**Dose-dependent effects of ISO on the viability and apoptosis of OGD-treated microglial cells in co-cultures.** Co-cultures with or without 3h-OGD stimulation were exposed to RA with or without ISO (0.7%, 1.4%, and 2.1%) for 30 min. After co-cultures were continuously cultured for 24 h under normal conditions, microglial cells were collected for further analyses. (**A**) MTT assay shows microglial cell viability. (**B**) Quantification of apoptotic microglial cells under the indicated treatments by Flow cytometry. (**C**) Flow cytometric analysis showing percentage of Annexin V^+^ PI^+^ apoptotic microglial cells under the indicated treatments. (**D**) Quantitative analysis of caspase-3 activity. Representative data are from three independent experiments and expressed as mean ± SD. Statistical significance: ^*^P < 0.05 vs. Ctrl groups; ^#^*P* < 0.05 vs. OGD + RA group. Ctrl: control; ISO: isoflurane; OGD: oxygen and glucose deprivation; RA: room air.

Since OGD induces BV-2 cell injury [19], we here investigated the effects of different doses of ISO (0.7, 1.4 and 2.1%) post-conditioning on OGD-exposed rat microglial cells. OGD reduced the viability (Figure 2A) and induced the apoptosis in microglial cells (Figure 2B–2D). 2.1%, 1.4%, and 0.7% ISO markedly decreased OGD-led microglial cell apoptosis, and 2.1% and 1.4% ISO showed little more protective effects than 0.7% ISO did (Figure 2A–2D). Since the clinical anesthetic dose of ISO (1.2%–2.5%) shows adverse effects in critically ill patients [23], we selected 0.7% ISO for further studies. These results demonstrate that 0.7% ISO abolishes OGD-caused microglial viability decline and apoptosis *in vitro*.

### Sub-anesthetic ISO post-conditioning represses OGD-induced COX2/PGE_2_ and iNOS/NO generation and inflammatory responses in rat microglial cells

**Figure 3:**
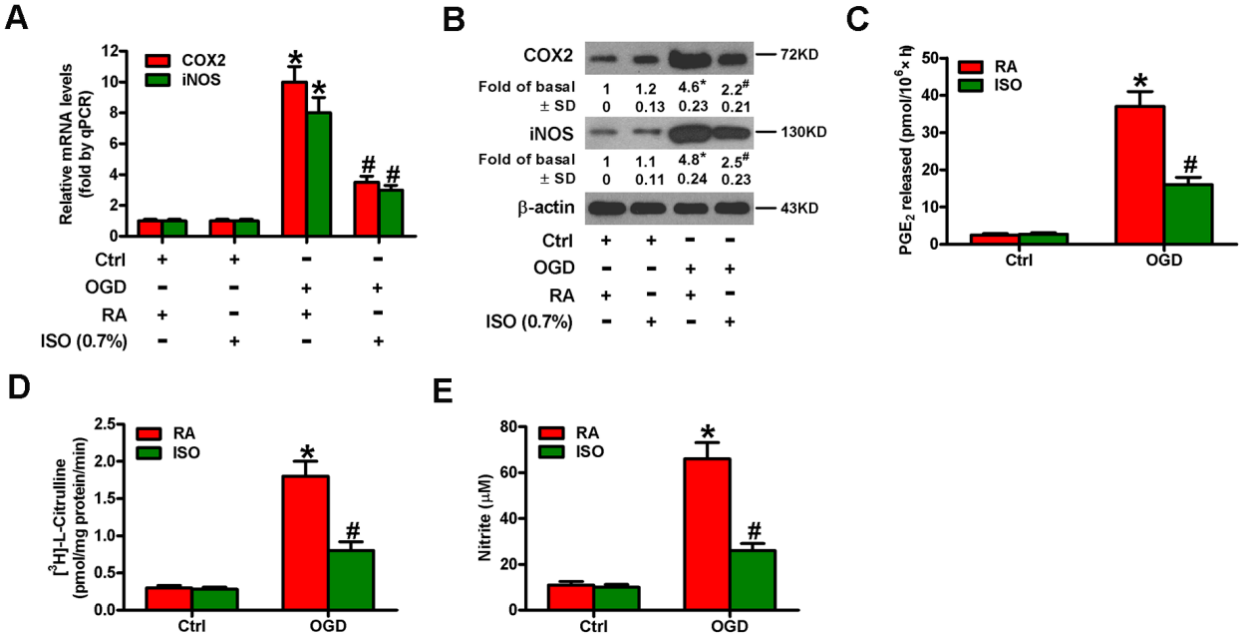
**Sub-anesthetic ISO post-conditioning abates OGD-induced COX2/PGE_2_ and iNOS/NO generation in microglial cells in co-cultures.** Co-cultures with or without 3 h-OGD stimulation were exposed to RA with or without 0.7% ISO for 30 min. And co-cultures were continuously cultured under normal conditions for 6, 12, or 24 h after OGD treatment. Then, microglial cells were harvested to measure some indexes. (**A**) qPCR analysis of COX2 and iNOS mRNA levels at 6 h after OGD treatment. GAPDH was used as the endogenous control. (**B**) Western blot analysis of COX2 and iNOS protein levels at 12 h after OGD treatment. β-actin was used as the internal control. (**C**) RIA analysis of PGE_2_ levels at 24 h after OGD treatment. (**D**) The iNOS activity was assessed by monitoring the conversion of arginine to citrulline at 12 h after OGD treatment. (**E**) Colorimetric estimation of NO levels with Griess reagent at 24 h after OGD treatment. Representative data are from three independent experiments and expressed as mean ± SD. Statistical significance: ^*^P < 0.05 vs. Ctrl groups; ^#^*P* < 0.05 vs. OGD + RA group. Ctrl: control; ISO: isoflurane; OGD: oxygen and glucose deprivation; RA: room air.

**Figure 4:**
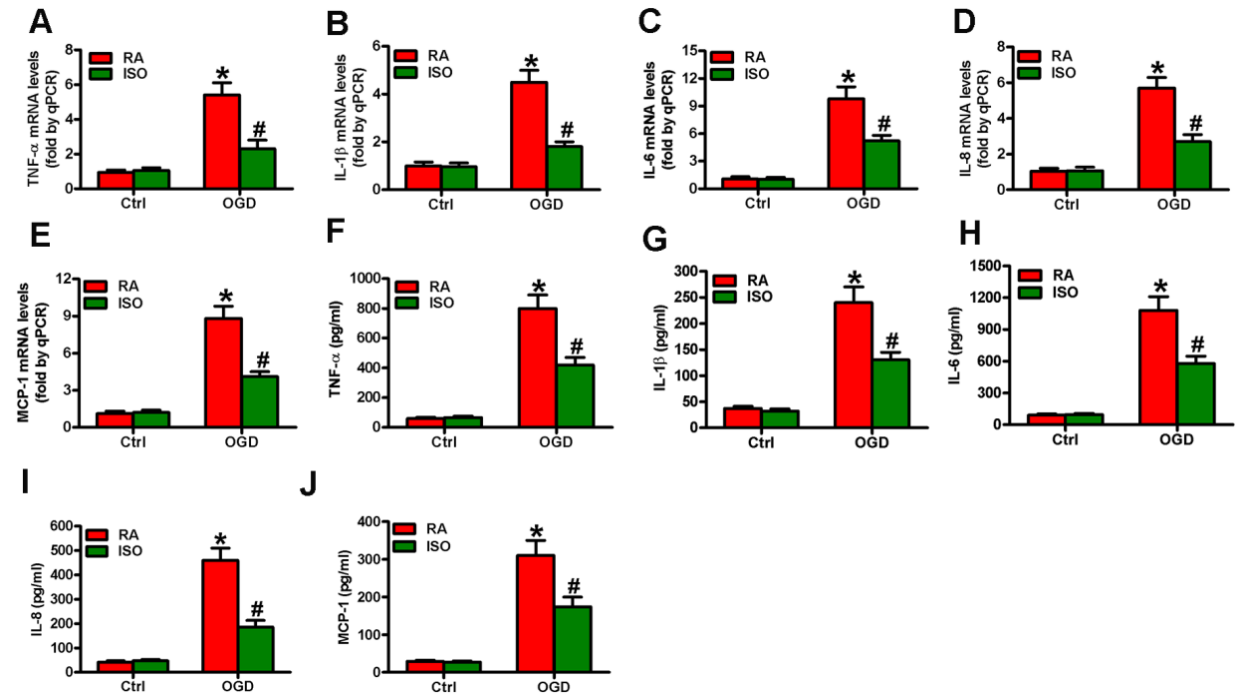
**Sub-anesthetic ISO post-conditioning reduces OGD-enhanced inflammatory factors in microglial cells in co-cultures.** Co-cultures with or without 3 h-OGD stimulation were exposed to RA with or without 0.7% ISO for 30 min. And co-cultures were continuously cultured under normal conditions for 6 or 12 h after OGD treatment. Then, microglial cells were harvested for next analyses. (**A**–**E**) qPCR analysis of (**A**) TNF-α, (**B**) IL-1β, (**C**) IL-6, (**D**) IL-8 and (**E**) MCP-1 mRNA levels at 6 h after OGD treatment. (**F**–**J**) ELISA analysis of (**F**) TNF-α, (**G**) IL-1β, (**H**) IL-6, (**I**) IL-8 and (**J**) MCP-1 protein levels at 12 h after OGD treatment. Representative data are from three independent experiments and expressed as mean ± SD. Statistical significance: ^*^P < 0.05 vs. Ctrl groups; ^#^*P* < 0.05 vs. OGD + RA group. Ctrl: control; ISO: isoflurane; OGD: oxygen and glucose deprivation; RA: room air.

Since OGD causes inflammatory responses in microglial cells [11], we tested the effects of 0.7% ISO on the expression of pro-inflammatory cytokines and chemokines in OGD-stimulated rat microglia. OGD enhanced COX2 and iNOS mRNA (Figure 3A) and protein (Figure 3B) levels, PGE_2_ release (Figure 3C), iNOS activity (Figure 3D), and NO production (Figure 3E) in rat microglial cells, which was impeded by 0.7% ISO (Figure 3A–3E). Furthermore, qPCR and ELISA assays showed that 0.7% ISO reduced OGD-induced mRNA (Figure 4A–4E) and protein (Figure 4F–4J) expressions of TNF-α, IL-1β, IL-6, IL-8, and MCP-1 in rat microglia. These data indicate that 0.7% ISO restrains OGD-induced inflammatory responses in rat microglial cells.

### Sub-anesthetic ISO post-conditioning inhibits OGD-induced NF-κB activation in rat microglial cells

**Figure 5:**
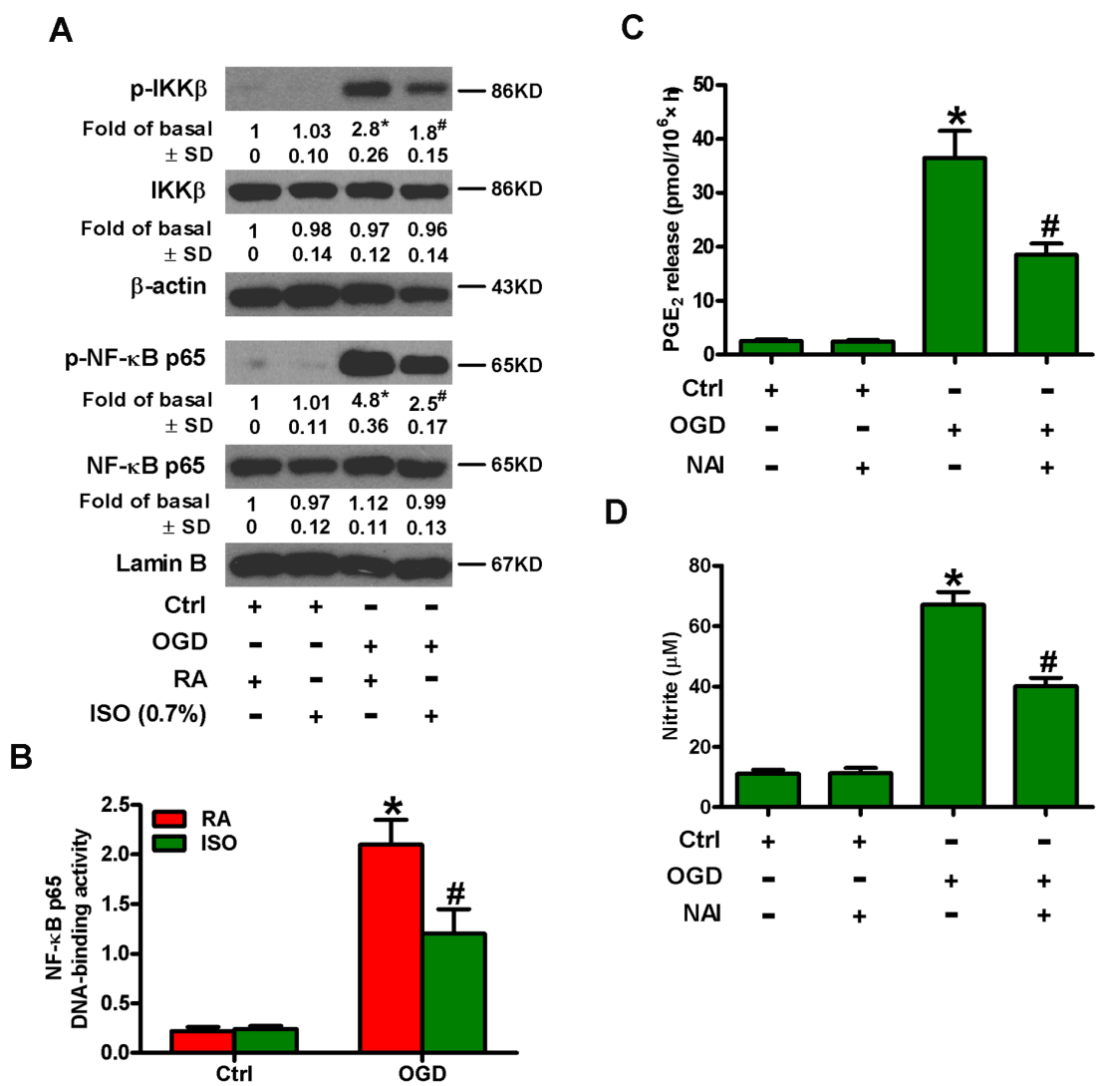
**Sub-anesthetic ISO post-conditioning inhibits OGD-led NF-κB p65 activation and PGE_2_ and NO production in microglial cells in co-cultures.** (**A**, **B**) At the end of 3 h-OGD or Ctrl treatment, co-cultures were exposed to RA with or without 0.7% ISO for 30 min. All the cells were continuously cultured under normal conditions for 6 or 12 h after OGD stimulation. Then, microglial cells were harvested for subsequent studies. (**A**) Representative western blots show total and phosphorylated IKKβ and NF-κB p65 levels at 6 h after OGD exposure. β-actin and lamin B were used as the internal controls. (**B**) NF-κB p65 DNA-binding activity was quantified using the TransAM NF-κB p65 transcription factor assay kit at 12 h after OGD insult. (**C**, **D**) Co-cultures with or without NAI (2 μM) pretreatment for 30 min were subjected to 3 h-OGD or Ctrl treatment and continuously cultured under normal conditions for 24 h after OGD stimulation. Then, microglial cells were collected for further analyses. (**C**) Quantification of PGE_2_ by RIA. (**D**) Quantification of NO production by Griess reagent. Representative data are from three independent experiments and expressed as mean ± SD. Statistical significance: ^*^P < 0.05 vs. Ctrl groups; ^#^*P* < 0.05 vs. OGD + RA or OGD group. Ctrl: control; ISO: isoflurane; OGD: oxygen and glucose deprivation; RA: room air.

NF-κB is an important transcription activator that regulates the expression of various inflammatory mediators, such as COX2 and iNOS [15, 16]. Therefore, we analyzed whether 0.7% ISO affected OGD-induced NF-κB activation. Western blot analysis revealed that 0.7% ISO reduced OGD-induced phosphorylation of IκB kinase β (IKKβ) and NF-κB p65 in microglial cells (Figure 5A). We further demonstrated that 0.7% ISO abated OGD-induced NF-κB DNA-binding activity in rat microglial cells by TransAM NF-κB DNA-binding ELISA assay (Figure 5B). We confirmed the central role of NF-κB by showing that pre-treating OGD-insulted microglial cells with NF-κB activation inhibitor (NAI) decreased PGE_2_ release (Figure 5C) and NO production (Figure 5D). These results illuminate that 0.7% ISO attenuates OGD-induced NF-κB activation in rat microglial cells.

### Sub-anesthetic ISO post-conditioning suppresses OGD-induced phosphorylation of p38 MAPK and subsequent NF-κB activation in rat microglial cells

**Figure 6:**
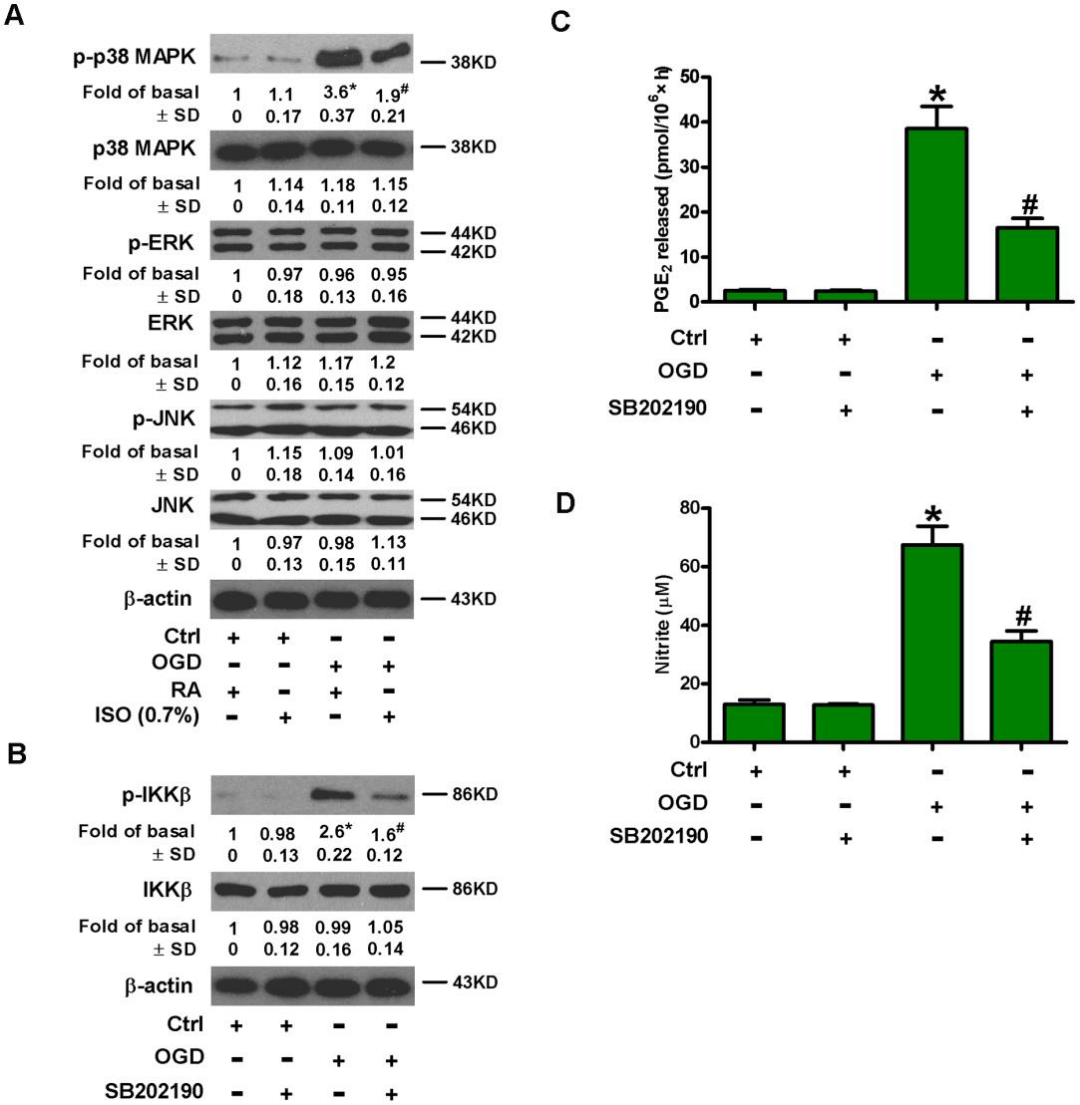
**Sub-anesthetic ISO post-conditioning hinders OGD-caused activation of p38 MAPK and NF-κB p65 in microglial cells in co-cultures.** (**A**) At the end of 3 h-OGD or Ctrl treatment, co-cultures were exposed to RA with or without 0.7% ISO for 30 min. All the cells were continuously cultured under normal conditions for 6 h after OGD exposure. Then, microglial cells were collected for further analyses. Representative western blots show protein expressions of p-p38 MAPK (Thr180/Tyr182), p38 MAPK, p-JNK1/2 (Thr183/Tyr185), JNK1/2, p-ERK1/2 (Thr185/Tyr187), and ERK1/2. β-actin was used as the internal control. (**B**–**D**) Co-cultures with or without SB202190 (10 μM) pretreatment for 30 min were subjected to 3 h-OGD or Ctrl treatment and continuously cultured under normal conditions for 6 or 24 h after OGD stimulation. Then, microglial cells were collected for further analyses. (**B**) Representative western blots show total and phosphorylated IKKβ levels at 6 h after OGD exposure. β-actin was used as the internal control. (**C**) Quantification of PGE_2_ levels by RIA at 24 h after OGD treatment. (**D**) Quantification of NO production by Griess reagent at 24 h after OGD challenge. Representative data are from three independent experiments and expressed as mean ± SD. Statistical significance: ^*^P < 0.05 vs. Ctrl groups; ^#^*P* < 0.05 vs. OGD + RA or OGD group. Ctrl: control; ISO: isoflurane; OGD: oxygen and glucose deprivation; RA: room air.

MAPK signaling activates NF-κB, which is essential for the induction of multiply inflammatory mediators [24]. We probed the effect of 0.7% ISO on phosphorylation of MAPKs in OGD-exposed microglial cells. OGD upregulated phospho-p38 MAPK, whereas, phosphorylated and non-phosphorylated ERK1/2 and JNK1/2 as well as p38 MAPK remained unchanged (Figure 6A). Moreover, 0.7% ISO reduced OGD-increased phospho-p38 MAPK in rat microglia (Figure 6A). Pretreatment with SB202190, a p38 MAPK inhibitor, hampered the OGD-induced phosphorylation of IKKβ (Figure 6B), release of PGE_2_ (Figure 6C) and production of NO (Figure 6D). These data demonstrate that 0.7% ISO hinders OGD-induced NF-κB activation in rat microglial cells by inhibiting p38 MAPK phosphorylation.

### Sub-anesthetic ISO post-conditioning hampers OGD-induced ROS generation in rat microglial cells

**Figure 7:**
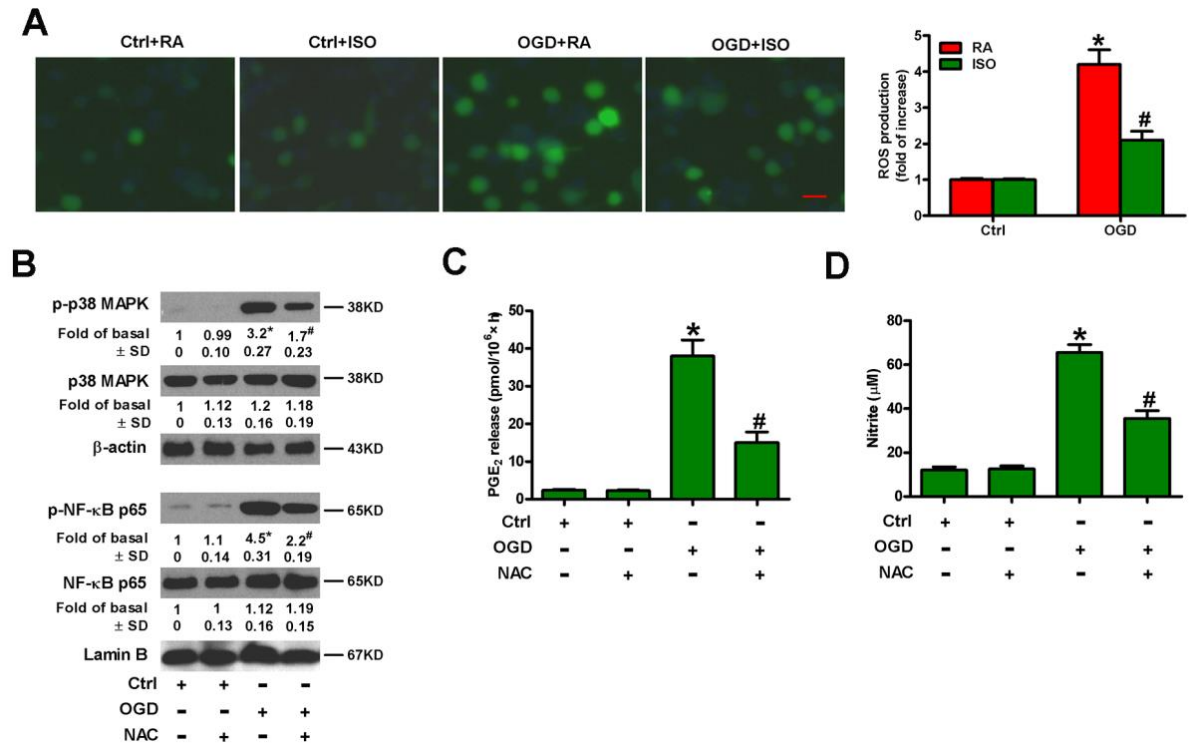
**Sub-anesthetic ISO post-conditioning represses ROS-mediated activation of p38 MAPK/NF-κB signaling in OGD-stimulated microglial cells in co-cultures.** (**A**) At the end of 3 h-OGD or Ctrl treatment, co-cultures were exposed to RA with or without 0.7% ISO for 30 min. All the cells were continuously cultured under normal conditions for 24 h after OGD exposure. Then, microglial cells were harvested for further analyses. The images of DCFH-DA-stained microglial cells were taken and ROS levels were calculated. Data represent the relative DCF fluorescence. Scale bar: 5 μm. (**B**–**D**) Co-cultures with or without NAC (5 mM) pretreatment for 30 min were subjected to 3 h-OGD or Ctrl treatment and continuously cultured under normal conditions for 6 or 24 h after OGD stimulation. Then, microglial cells were harvested for assays. (**B**) Representative western blots show total and phosphorylated p38 MAPK and NF-κB p65 levels at 6 h after OGD exposure. β-actin and lamin B were used as the internal controls. (**C**) Quantification of PGE_2_ levels by RIA at 24 h after OGD exposure. (**D**) Quantification of NO production by Griess reagent at 24 h after OGD stimulation. Representative data are from three independent experiments and expressed as mean ± SD. Statistical significance: ^*^P < 0.05 vs. Ctrl groups; ^#^*P* < 0.05 vs. OGD + RA or OGD group. Ctrl: control; ISO: isoflurane; OGD: oxygen and glucose deprivation; RA: room air; NAC: N-acetyl-L-cysteine.

OGD leads to oxidative stress in microglia [25]. ISO reduces ROS production in zymosan-insulted murine Kupffer cells [17]. Thus, we addressed whether 0.7% ISO repressed OGD-induced ROS signaling in microglial cells. As shown in Figure 7A, 0.7% ISO reduced OGD-enhanced ROS generation in microglial cells. ROS is essential for inflammatory promotion because it increases the activation of p38 MAPK and downstream NF-κB in Kupffer cells [17]. Pretreatment of rat microglial cells with N-acetyl-L-cysteine (NAC) reduced OGD-induced phosphorylation of p38 MAPK and NF-κB p65 (Figure 7B), PGE_2_ release (Figure 7C) and NO production (Figure 7D). These results suggest that 0.7% ISO suppresses ROS-mediated p38 MAPK/NF-κB signaling in OGD-challenged rat microglial cells.

### Sub-anesthetic ISO post-conditioning lessens OGD-triggered microglial cell apoptosis in a ROS- or NO-dependent fashion

**Figure 8:**
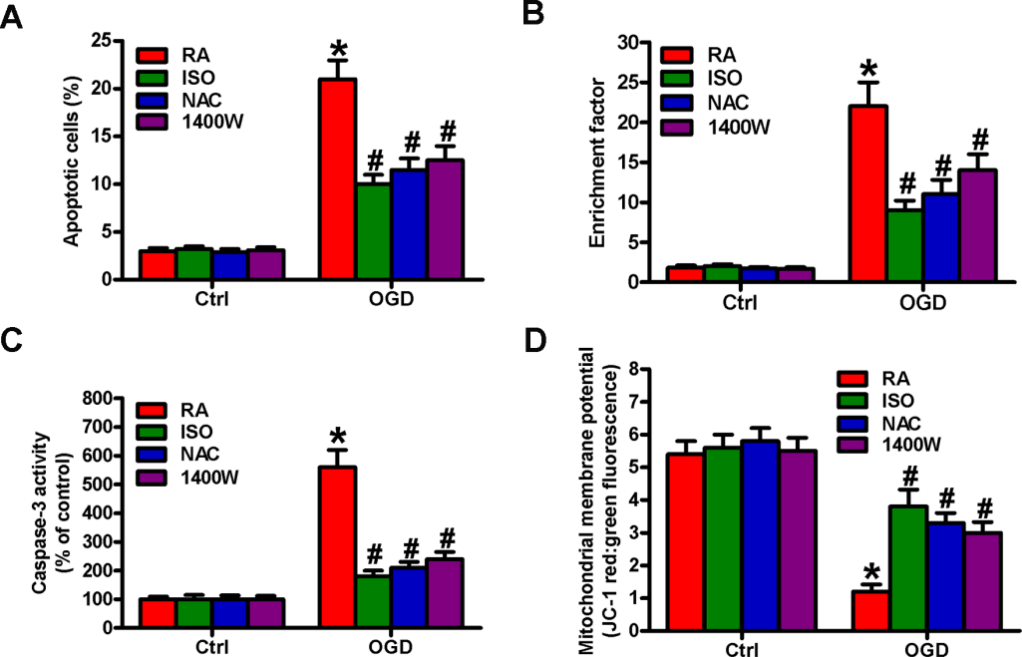
**Sub-anesthetic ISO post-conditioning hampers ROS- or NO-mediated OGD-exposed microglial cell apoptosis in co-cultures.** Co-cultures with or without NAC (5 mM) or 1400 W (50 μM) pretreatment for 30 min were stimulated with OGD or Ctrl for 3 h and subsequently exposed to RA with or without 0.7% ISO for 30 min. All the cells were continuously cultured under normal conditions for 24 h after OGD stimulation. Then, microglial cells were collected for further analyses. (**A**) Flow cytometry analysis of microglial cell apoptosis. (**B**) The nucleosomal fragmentation assay for assessing microglia apoptosis. (**C**) Quantitative analysis of caspase-3 activity. (**D**) Flow cytometry analysis of JC-1 stained microglial cells. Representative data are from three independent experiments and expressed as mean ± SD. Statistical significance: ^*^P < 0.05 vs. Ctrl groups; ^#^*P* < 0.05 vs. OGD + RA group. Ctrl: control; ISO: isoflurane; OGD: oxygen and glucose deprivation; RA: room air; NAC: N-acetyl-L-cysteine.

OGD results in BV-2 cell apoptosis [19]. We observed the effects of 0.7% ISO on the apoptosis of OGD-treated microglial cells. Flow cytometry analysis showed that 0.7% ISO decreased OGD-induced microglial cell apoptosis (Figure 8A). Moreover, 0.7% ISO reduced OGD-enhanced nucleosomal fragmentation (Figure 8B) and caspase-3 activity (Figure 8C) in microglia. The low mitochondrial membrane potential was recovered by 0.7% ISO in OGD-challenged microglial cells (Figure 8D). ROS and NO are highly involved in microglial cell apoptosis [7, 8]. Pretreatment with NAC or 1400W significantly reduced OGD-elevated apoptotic microglial cells (Figure 8A–8D), in parallel with 0.7% ISO. These data imply that 0.7% ISO suppresses OGD-induced microglial cell apoptosis by inhibiting ROS and NO production.

### Sub-anesthetic ISO post-conditioning enhances the viability and apoptosis resistance in OGD-insulted neurons *in vitro*

**Figure 9:**
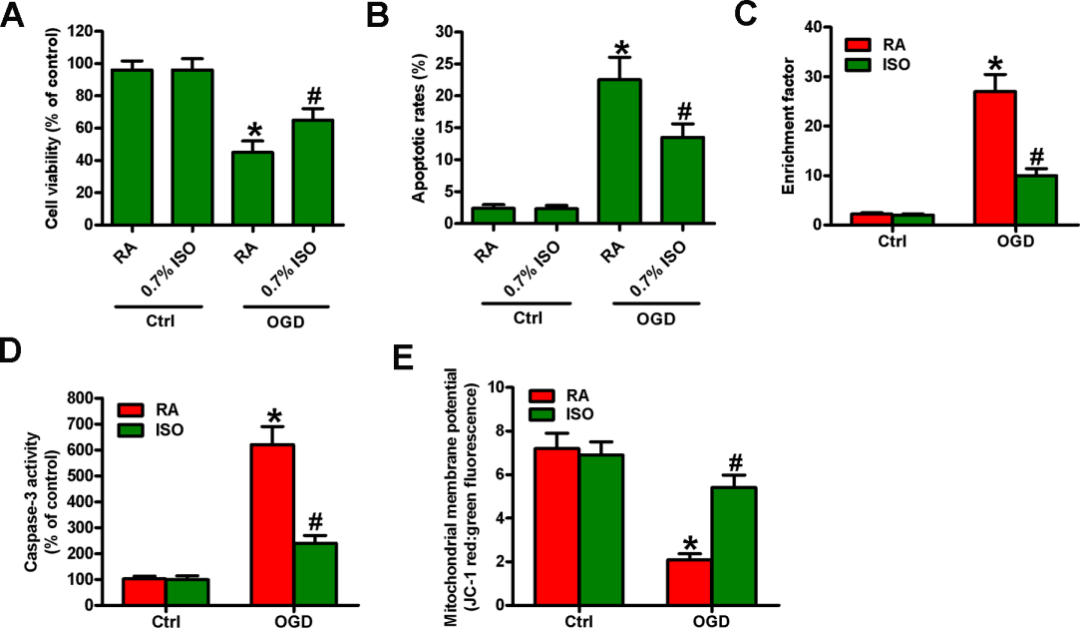
**Sub-anesthetic ISO post-conditioning increases the survival of OGD-treated neurons in co-cultures.** Co-cultures with or without 3 h-OGD stimulation were exposed to RA with or without 0.7% ISO for 30 min. After co-cultures were continuously cultured for 24 h under normal conditions, neurons were collected for further analyses. (**A**) MTT assay shows neuronal viability. (**B**) Flow cytometry analysis of neuronal apoptosis. (**C**) The nucleosomal fragmentation assay for assessing neuron apoptosis. (**D**) Quantitative analysis of caspase-3 activity. (**E**) Flow cytometry analysis of JC-1 stained neurons. Representative data are from three independent experiments and expressed as mean ± SD. Statistical significance: ^*^P < 0.05 vs. Ctrl groups; ^#^*P* < 0.05 vs. OGD + RA group. Ctrl: control; ISO: isoflurane; OGD: oxygen and glucose deprivation; RA: room air.

Reportedly, OGD induces neuronal injury [26]. Thus, we explored the effects of 0.7% ISO on OGD-treated rat neurons. As depicted in Figure 9A, OGD markedly decreased neuronal viability, which was counteracted by 0.7% ISO. Moreover, 0.7% ISO significantly reduced OGD-induced enhancement in neuronal apoptosis (Figure 9B–9E). These results reveal that 0.7% ISO increases the survival in OGD-challenged neurons *in vitro*.

### Sub-anesthetic ISO post-conditioning retards the inflammation and apoptosis in MCAO rat brains

**Figure 10:**
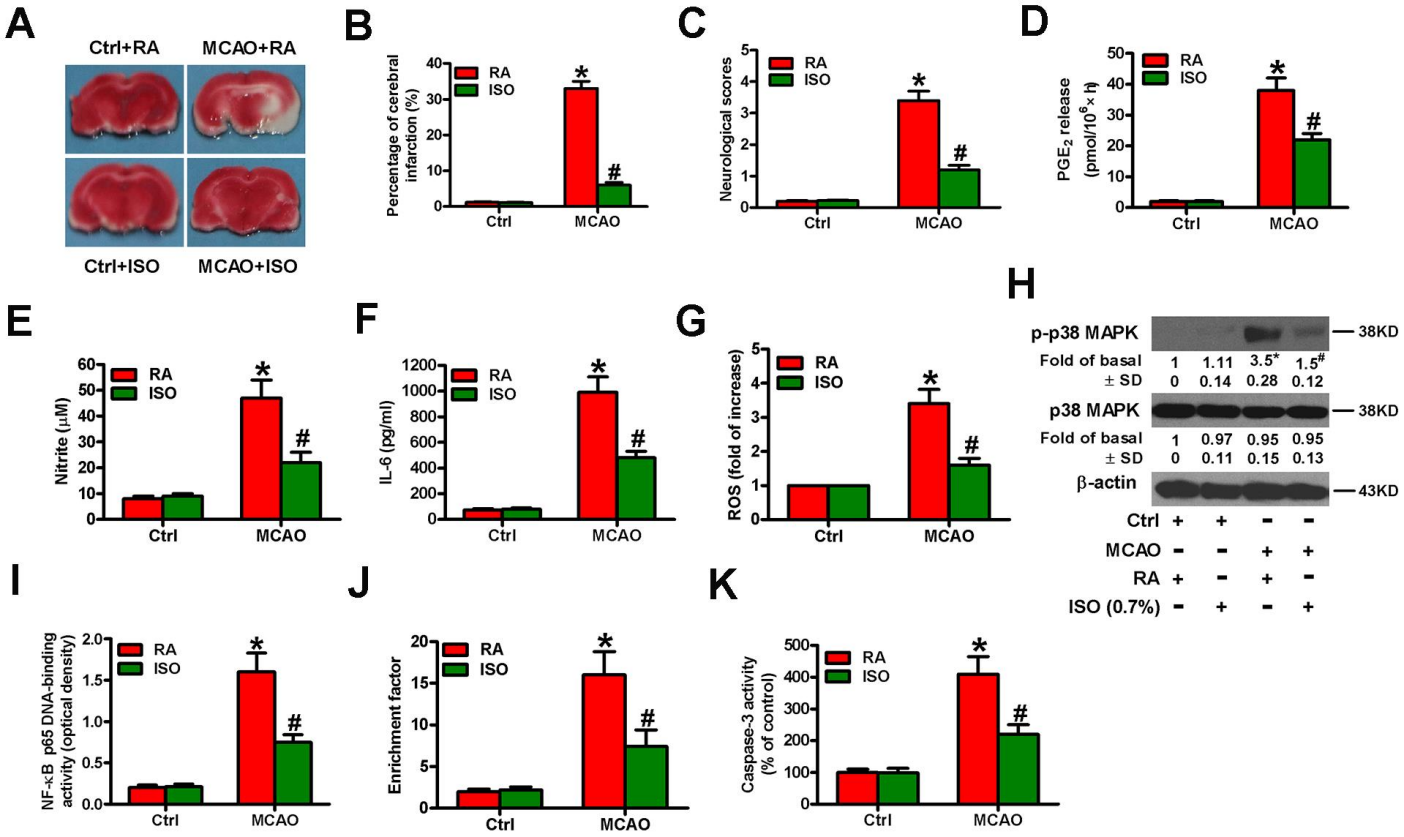
**Sub-anesthetic ISO post-conditioning attenuates MCAO-induced inflammation and apoptosis in rat brains.** Rats were subjected to a right MCAO or sham operation (control) for 2 h and then treated with or without 0.7% ISO for 1 h. At 24 h after MCAO, seventy-six rats (Ctrl + RA, *n* = 20; Ctrl + ISO, *n* = 20; MCAO + RA, *n* = 17; MCAO + ISO, *n* = 19) still survived and then they were sacrificed under anesthesia. (**A**) Representative images of the TTC-stained brain tissue sections showing infarction areas (*n* = 8 per group). (**B**) Quantitative measurements of the infraction volume (*n* = 8 per group). (**C**) Histogram plots show neurologic deficit scores (*n* = 12 per group). (**D**) Quantification of rat serum PGE_2_ levels by RIA (*n* = 16 per group). (**E**) Quantification of rat serum NO levels by Griess reagent (*n* = 16 per group). (**F**) ELISA analysis of the rat serum IL-6 levels (*n* = 8 per group). (**G**) Analysis of ROS level by DCFH-DA assay in brain homogenates (*n* = 9 per group). The data represent the relative DCF fluorescence. (**H**) Representative western blots show total and phospho-p38 MAPK in the brain homogenates (*n* = 9 per group). β-actin was used as the normal control. (**I**) Estimation of NF-κB p65 DNA-binding activity in brain homogenates using a TransAM NF-κB p65 transcription factor assay (*n* = 9 per group). (**J**) Estimation of nucleosomal fragmentation in brain tissues (*n* = 9 per group). (**K**) Quantitative measurement of caspase-3 activity (*n* = 9 per group). Representative data are from three independent experiments and expressed as mean ± SD. Statistical significance: ^*^P < 0.05 vs. Ctrl groups; ^#^*P* < 0.05 vs. OGD + RA group. Ctrl: control; ISO: isoflurane; MCAO: middle cerebral arterial occlusion; RA: room air.

Next, we examined the effects of 0.7% ISO on the brain injuries of MCAO rats. We found that 0.7% ISO reduced infarct volume (Figure 10A, 10B) and neurologic deficit scores (Figure 10C) and serum levels of PGE_2_ (Figure 10D), NO (Figure 10E), and IL-6 (Figure 10F) in MCAO rats. As shown in Figure 10G, ROS levels were considerably reduced by 0.7% ISO in the brain homogenates of MCAO rats. Western blot analysis showed that 0.7% ISO decreased phosphorylation of p38 MAPK in the MCAO rat brains (Figure 10H). Moreover, 0.7% ISO inhibited NF-κB p65 DNA-binding activity in the brains of MCAO rats (Figure 10I). 0.7% ISO also reduced the apoptosis in the brains of MCAO rats by diminishing nucleosomal fragmentation (Figure 10J) and caspase-3 activity (Figure 10K). These data indicate that 0.7% ISO ameliorates the brain injuries of MCAO rats.

## DISCUSSION

In this study, 0.7% ISO post-conditioning reduces the inflammation and apoptosis in OGD-stimulated neuron/microglia co-cultures *in vitro* and the brain injuries of MCAO rats *in vivo*. Several key findings are as follows. First, 0.7% ISO increased the viability of OGD-exposed microglial cells. Second, 0.7% ISO decreased the expression and activity of COX2 and iNOS, and lowered the release of PGE_2_ and NO production in OGD-stimulated microglial cells and MCAO rat brains. Third, 0.7% ISO reduced the production of pro-inflammatory cytokines and chemokines by inhibiting ROS-mediated activation of p38 MAPK/NF-κB signaling in OGD-challenged microglial cells and MCAO rat brains. Fourth, 0.7% ISO abated OGD-induced microglial cell apoptosis in a ROS- or NO-dependent manner. Fifth, 0.7% ISO enhanced the viability and lessened the apoptosis in OGD-challenged primary rat neurons. Last, 0.7% ISO reduced brain injuries in MCAO rats. These findings suggest that 0.7% ISO post-conditioning effectively protects against hypoxic-ischemic brain injury partly by inhibiting the inflammation or apoptosis of rat microglia and neurons.

In the CNS, neuron and microglia communicate with each other and form a functional unit. Altering either of them may change the status or function of the others. Thus, co-culturing this functional unit may better mimic the pathological situation *in vivo* and address the notion that the anti-inflammatory response of therapeutic agents may confer neuroprotection thoroughly [27]. Hypoxia-ischemia induces the hyper-activation of microglial cells, which eventually causes neuronal injury [27]. Wang et al. [28] reported that the suppression of microglial activation may be neuroprotective against ischemia. During cerebral ischemia, activated microglial cells migrate to inflammatory sites and produce excessive neurotoxic and inflammatory mediators, such as TNF-α, IL-1β, IL-6, MCP-1, MIP-1, PGE_2_ and NO, which exacerbate neuronal damage [29, 30]. Although the significant enhancement of neurotoxins appeared in OGD-stressed neurons, it is a very small amount as compared to activated microglia [29]. Thus, the reduction of neural cell death in ischemic penumbra was primarily attributed to the targeted inhibition of inflammatory responses in activated microglia [10]. In this study, OGD intensely induced microglia activation, which produces various neurotoxins, such as NO, oxygen radicals, arachidonic acid derivatives, and cytokines, contributing to neuronal injury and apoptosis. The literature to date reports the activation of several signaling pathways in microglia after ischemia and include the following: the IKK-NF-κB pathway, typically activated by oxidative and inflammatory stress [31]; the p38 MAPK pathways that are activated by cytokines and growth factors [32, 33]. Activation of these pathways typically results in the release of pro-inflammatory cytokines and nitric oxide. Generally, the inhibition of these pathways is neuroprotective [32]. The anesthetic agent, isoflurane (ISO), exhibits anti-oxidative and anti-inflammatory effects [15, 16]. 2% ISO protects against OGD-caused microglia injury by inhibiting the activation of MAPK/NF-κB signaling [19]. However, clinical use of ISO at anesthetic concentrations (1.2%-2.5%) is limited due to adverse systemic effects. Previously, we demonstrated that 0.7% ISO protects against zymosan-induced systemic inflammatory response syndrome by upregulating antioxidant enzymes and reducing inflammatory mediators [17, 34]. We pretreated zymosan-insulted neutrophils with the specific inhibitors of ROS (NAC), p38 MAPK (SB202190), NF-κB p65 (NAI), ONOO^−^ (FeTPPS) and found that 0.7% ISO reduced zymosan-induced neutrophil inflammatory responses by targeting ROS/p38 MAPK/NF-κB p65/iNOS signaling [18]. Therefore, we investigated the mechanism of action of 0.7% ISO post-conditioning in OGD-challenged co-cultured microglia and neurons and MCAO rat brains. We found that 0.7% ISO reduced OGD-induced inflammation in microglia by inhibiting the phosphorylation of IKKβ and NF-κB p65 and subsequent nuclear translocation and DNA-binding of NF-κB p65. 0.7% ISO repressed ROS generation and ROS-mediated activation of p38 MAPK/NF-κB signaling and abated neuron apoptosis. Intriguingly, the *in vivo* results of MCAO rat brains were partially consistent with *in vitro* findings, as evidenced by decreased ROS, p-p38 MAPK, and p-NF-κB and apoptosis. These observations are partly attributed to the beneficial effects of ISO on ischemia-activated microglia. However, further studies are required to fully elucidate the association between *in vivo* rat brain injuries and microglial cell activation in the protective effect mediated by ISO. Previous studies have demonstrated that neuroprotection by merely targeting one specific pathway of the ischemic cascade is incomplete [35, 36]. Treatment strategies that target multiple mechanisms are therefore important for the successful reduction of ischemic brain injury. With the protective potency and multiple-targeting capacity of ISO, it is speculated that ISO post-conditioning conferred significant neuroprotection against ischemic brain injury by both indirect (anti-microglia inflammation) and direct (anti-neuron death) mechanisms. Nevertheless, further evaluation to reveal a benefit of ISO is necessary to draw a firm conclusion.

In certain settings, microglia produce anti-inflammatory cytokines [37] and are neuroprotective during ischemia [38]. Depletion of microglial cells exacerbates stroke, whereas, injection of microglial cells improves stroke symptoms, thereby suggesting that tissue-resident microglia protect against ischemic brain injury [39]. Loss of microglial cells also aggravates ischemia-induced local inflammation and injury [40]. Microglia apoptosis plays a critical role in the pathophysiology of cerebral ischemia-reperfusion injury, which is mediated by enhanced ROS levels [7]. OGD induces microglial apoptosis by activating ROS-dependent p53 and PI3K/Akt pathways [41]. Microglial activation results in the excessive production of NO, and NO promotes microglial apoptosis in an autocrine manner [8]. Overall, pathogenesis of ischemic brain injury involves microglial apoptosis due to oxidative/nitrosative stress [42]. We demonstrated that sub-anesthetic ISO reduced the apoptosis in the OGD-insulted microglial cells and the brains of MCAO rats, similar to treatment with NAC (anti-oxidant) or 1400W (iNOS inhibitor), suggesting that ISO suppresses microglial apoptosis in a ROS- or NO-dependent manner. Cerebral ischemia triggers microglial apoptosis by modulating a variety of death signals including the caspase-dependent apoptotic pathway [43]. Caspase-3 is a pivotal executor of the intrinsic apoptotic signaling and mediates the processing of apoptosis-associated proteins and DNA fragmentation [44]. Herein, we illuminated that sub-anesthetic ISO abrogated the apoptosis in the OGD-challenged microglial cells and the brains of MCAO rats possibly by inhibiting the ROS/NO-dependent apoptotic pathway that includes caspase-3.

This study has several limitations. First, we did not perform time-dependent effects of 0.7% ISO on pro- and anti-inflammatory factors to comprehend the molecular mechanisms in greater detail. Second, we did not address the *in vivo* application of NF-κB and p38 MAPK inhibitors as well as ROS scavengers and monitor their effects on the inflammatory parameters and subsequently brain damage. Third, we further needed to investigate how to interact between microglia and neurons and that what interaction affected the OGD- or MCAO-induced inflammation and apoptosis. Fourth, more investigation is required to confirm that inflammatory mediators are mainly derived from microglia but not neurons. Fifth, whether the anti-inflammation and anti-apoptosis effects of 0.7% ISO *in vivo* are achieved through microglia is not fully determined. Finally, limitations including aging, types of animal, treatment protocol, and the timing periods of administration are critical for evaluating the therapeutic effects of sub-anesthetic ISO.

In conclusion, we demonstrate that sub-anesthetic ISO post-conditioning decreases the inflammation and apoptosis in OGD-insulted microglia in co-cultures partly by blocking ROS/p38 MAPK/NF-κB signaling or inhibiting ROS- or NO-dependent pathways, respectively. 0.7% ISO reduces the apoptosis of OGD-exposed neurons in co-cultures. *In vivo* results with MCAO rats were partly consistent with the *in vitro* findings. This study reveals the therapeutic potential of sub-anesthetic ISO in ischemic brain injuries.

## MATERIALS AND METHODS

### Reagents

ISO was purchased from Baxter Healthcare Corporation (Deerfield, IL, USA). Primary antibodies, including rabbit anti-rat JNK (cat#: ab76125), p38 MAPK (cat#: ab170099), ERK 1/2 (cat#: ab184699), IKKβ (cat#: ab124957), NF-κB p65 (cat#: ab16502), COX2 (cat#: ab15191), iNOS (cat#: ab3523), β-actin (cat#: ab179467), lamin B (cat#: ab16048), and p-NF-κB p65 (Ser536; cat#: ab76302) were acquired from Abcam (Cambridge, UK). Rabbit anti-rat phosphorylated (p)-JNK (Thr183/Tyr185; cat#: 4668), p-p38 MAPK (Thr180/Tyr182; cat#: 4511), p-ERK1/2 (Thr202/Tyr204; cat#: 4370), and p-IKKβ (Ser180; cat#: 3671) antibodies were procured from Cell Signaling Technology, Inc. (Beverly, MA, USA). Horseradish peroxidase (HRP)-conjugated anti-rabbit IgG (cat#: 0111-01) was provided by Chemicon (Temecula, CA, USA). NF-κB activation inhibitor (NAI; cat#: S1014-67.1) and p38 MAPK activation inhibitor (SB202190; cat#: 152121-30-7) were supplied by Biomol (Plymouth Meeting, PA, USA). ROS scavenger (NAC; cat#: A7250) and iNOS activity inhibitor (1400W; cat#: W4262) were purchased from Sigma–Aldrich (St. Louis, MO, USA). All the other reagents were obtained from Sigma unless specially stated. All suspensions were freshly prepared before use.

### Animals and ethics statement

One-day-aged and 8-week-old (weighing 280 g to 300 g) male Sprague-Dawley (SD) rats were purchased from Laboratory Animal Center of Henan Province (Zhengzhou, Henan, China) and maintained at 22° C-24° C with a regular 12 h/12 h day/night cycle. Standard laboratory chow and tap water was acquired *ad libitum*. All experimental procedures were approved by the Institutional Animal Research Ethics Board of the 150^th^ Central Hospital of PLA (Ethics number: 150-2016-029) and conducted in full compliance with the criteria for the care and use of laboratory animals laid out by the National Institutes of Health (Bethesda, MD, USA). Euthanasia was performed according to American Veterinary Medical Association guidelines on euthanasia (June 2007) by using sodium pentobarbital (Sigma; cat#: P-010).

### Preparation of MCAO model and ISO administration

The rat MCAO model was established as previously described with slight modifications [45]. The rats were anesthetized with sodium pentobarbital. Right MCAO was achieved by advancing a 3-0 monofilament nylon suture with a rounded tip (Beijing Sunbio Biotech Co. Ltd., Beijing, China) into the right internal carotid artery via the external carotid artery until slight resistance was encountered. The common carotid artery was transiently occluded but was not ligated during the process. At 2 h after the onset of MCAO, the rats were re-anesthetized and the nylon suture was removed. In the sham groups, the nylon suture was placed in an identical fashion but without actually occluding the vessel or undergoing hypoxia. In the ISO groups, the rats were placed in a sealed Plexiglas chamber with inflow and outflow outlets and allowed to inhale ISO for 1 h after MCAO, as previously described [15, 16]. The animals without ISO administration were exposed to room air (RA) in the chamber as the control. Blood samples and brain tissues were collected and some indexes were measured at the indicated time points.

### *In vivo* experimental grouping

Eighty male SD rats were randomly divided into four groups (*n* = 20 per group). (1) Control (Ctrl) + RA group: rats underwent sham operation and then inhaled RA (vehicle) for 1 h. (2) Ctrl + 0.7% ISO group: rats were subjected to sham operation and subsequently received 1 h of ISO inhalation. (3) MCAO + RA group: rats were subjected to MCAO for 2 h and allowed to inhale RA for 1 h after MCAO. (4) MCAO + 0.7% ISO group: rats suffered MCAO for 2 h followed by 1 h of ISO inhalation.

### Assessment of brain damage in MCAO rats

Brain infarct volume was assessed by 2,3,5-triphenyltetrazolium chloride (TTC; Sigma; cat#:17779) staining. At 24 h after MCAO or sham treatments, the rats were euthanized with sodium pentobarbital. The brains were then rapidly removed and coronally sliced into 2 mm thickness. The sections were incubated with a 2% solution of TTC for 30 min at 37° C and fixed with 10% formaldehyde (Sigma; cat#: F1635). Unstained areas were indicative of infarct regions, whereas red-stained sections represented the normal tissues. The slides were photographed and the infarct size of each section was quantified by using a computerized image analysis system (NIH Image v.1.61, USA). Total lesion volume was calculated as total infarct area in each section multiplied by the distance between sections.

### Neurobehavioral functional scoring

The neurological deficit scores of various groups were blindly examined by an experienced investigator. Neurological deficit grading was determined according to the five-point scale: grade 0, no neurologic deficit; grade 1, failure to extend left forepaw fully; grade 2, circling to the left; grade 3, falling to the left; grade 4, unable to walk spontaneously and depression of consciousness.

### Primary rat microglia and neuron isolation and co-culture

Rat microglial cells were isolated from one-day-old SD rats and cultured as previously described [46]. The brains were rapidly removed after euthanizing one-day-aged rats with sodium pentobarbital. The cerebral cortex was dissected and the meninges were separated. The cortex tissue was minced and the single cell suspension was prepared by incubation with 0.25% Trypsin in 1 mM of ethylenediamine tetraacetic acid (EDTA) for 30 min at 37° C. After washing twice with D-Hank’s solution, cortical cells were grown in DMEM/F12 medium (Gibco, BRL, UK; cat#: 11320033) at a concentration of 1 × 10^6^ cells/ml in uncoated culture flasks at 37° C and 5% CO_2_ for 30 min. The cells were then transferred to a new culture flask to eliminate fibroblasts that stick to the uncoated flasks. The culture medium was refreshed every 3 days for 10−14 days following which the microglial cells were separated from the astrocyte monolayer by gently agitating the flasks at 37° C in a shaker-incubator at 180 rpm. The identity and purity of microglia were determined by immunofluorescent staining with CD11b (> 98%; Abcam; cat#: ab8878). To distinguish microglia from macrophages, we performed the double immunofluorescent staining with CD11b and CD45 (Abcam; cat#: ab10558). Cortical neuron-enriched cultures were obtained as previously reported [47]. The identity and purity of the primary neurons was confirmed by immunostaining with NeuN, the neuron-specific marker (> 90%; Abcam; cat#: ab177487). Prior to all experiments, more than 99% of the cells were determined viable using Live/Dead violet (Invitrogen, Carlsbad, CA, USA; cat#: L34963).

Co-culture of the rat primary microglia and neurons was established as previously described with some modification [48]. The primary microglia (lower chamber) and neurons (upper chamber) were co-cultured for 24 h in a Transwell co-culture system with non-contact inserts (Corning Life Sciences, Tewksbury, MA, USA), sharing the DMEM/F12 medium supplemented with 2% B27 and 1% penicillin/streptomycin through a 0.4 μm transmembrane that prevents cell migration, but allows small molecule exchange. Moreover, the images of cell morphology under Ctrl and OGD treatments were taken as the Supplementary Figure 1.

### *In vitro* experimental procedure

Co-cultured microglia and neurons were randomly allocated into four groups (*n* = 3). (1) OGD + RA group: co-cultures were grown in glucose-free medium for 3 h in oxygen-free N_2_/CO_2_ (95%/5%) gas at 37° C and then incubated in a normoxic incubator with normal culture medium for the indicated time durations. (2) OGD + ISO group: co-cultures were cultivated in glucose-free medium for 3 h in oxygen-free N_2_/CO_2_ (95%/5%) gas at 37° C followed by inhalation of 0.7%, 1.4% or 2.1% ISO for 30 min in a metabolic chamber (Columbus Instruments, Columbus, OH, USA). ISO concentration was continuously monitored by sampling the exhaust gas with a Datex Capnomac (SOMA Technology Inc., Cheshire, CT, USA). Then, the co-cultures were incubated with normal culture medium and normoxia for the indicated time durations. (3) Ctrl + RA group: co-cultures were incubated with normal culture medium and normoxia. (4) Ctrl + ISO group: co-cultures were grown in normal culture medium and exposed to 30 min of 0.7%, 1.4% or 2.1% ISO.

Microglial cells were pretreated with or without 2 μM NAI, 10 μM SB202190, 5 mM NAC or 50 μM 1400W for 30 min, washed out, and subjected to the above treatments for the indicated time periods.

### Microglia and neuron viability assay

The viability of the co-cultured microglia and neuron was measured by using the 3-(4,5-dimethyldiazol-2-yl)-2,5-diphenyltetrazolium bromide (MTT; Sigma; cat#: M2128) assay. At 24 h after co-cultures were reoxygenated, 100 μl of MTT (5 mg/ml) solution was added to each well of co-cultures and incubated for 4 h at 37° C. Then, the supernatants were removed, and microglia remained in the previous plate, whereas neurons were transferred into another plate. 150 μl of dimethyl sulfoxide (Sigma; cat#: D8418) was added to each well, and the plates were immediately read at 490 nm on a scanning multi-well spectrometer (Model 550, Bio-Rad, Hercules, CA, USA).

### Annexin V-propidium iodide (PI) apoptosis assay

Microglia and neuron apoptosis was detected by staining with Annexin V-fluorescein isothiocyanate (FITC) and PI Kit (BD Biosciences, San Jose, CA, USA; cat#: 556420). At 24 h after co-cultures were reoxygenated, microglia and neurons were detached by trypsin and centrifuged at 2000 × *g* for 5 min. The cell pellets from different groups were resuspended in Annexin V-binding buffer at a concentration of 1 × 10^5^ cells/ml (microglia) and 1 × 10^4^ cells/ml (neurons). Then, 100 μl of cell suspension was stained with 10 μl of ready-to-use Annexin V-FITC at 37° C for 15 min and counterstained with 5 μl of PI in the dark for 30 min. The assays were conducted with a BD FACS Calibur flow cytometer (BD Biosciences) and the percentage of double positive apoptotic cells (Annexin V^+^ PI^+^) were determined by using the CellQuest software (BD Biosciences).

### Quantification of cytokine and chemokine by enzyme-linked immunosorbent assay (ELISA)

At 12 h after the rats underwent MCAO or the microglial cells were subjected to OGD treatment, the levels of TNF-α (cat#: PRTA00), IL-1β (cat#: PRLB00), IL-6 (cat#: PR6000B), IL-8 (cat#: PD8000C), and MCP-1 (cat#: DY3144-05) in the serum or microglial cell culture supernatants were measured by using commercially available ELISA kits (R&D Systems, Minneapolis, MN, USA) as per the manufacturers’ instructions. Optical density was determined using an ELISA plate scanner (CA94089, Molecular Devices, Sunnyvale, Canada).

### Measurement of PGE_2_ production

At 24 h after the rats underwent MCAO or the microglial cells were subjected to OGD treatment, the levels of PGE_2_ in the serum or microglial cell culture supernatants were measured by using a radioimmunoassay (RIA) kit (Amersham, Freiburg, Germany) following the manufacturer’s protocol.

### Quantitative real-time PCR (qPCR)

At 6 h after the microglial cells were treated with OGD, total RNA was extracted from 1 × 10^6^ cells by using TRIzol reagent (Invitrogen, Carlsbad, CA, USA; cat#: 15596018). The collected RNA was diluted to a concentration of 1 μg/μl and used to generate complementary DNA using a reverse transcription kit (Promega; cat#: A6002). qPCR assay was performed in Mastercycler® ep realplex (Eppendorf, Hamburg, Germany) using a standard IQTM SYBR Green Supermix kit (Bio-Rad, Berkeley, USA; cat#: 1708882). GAPDH was used as endogenous control. Relative mRNA levels were calculated using the 2^–ΔΔCt^ method [49]. The primer sequences were listed in Table 1.

**Table 1:** Primers used for qPCR assays.

**Gene**	**Primer sequences (5′ to 3′)**	
TNF-α	Forward	TTGACCTCAGCGCTGAGTTG
	Reverse	CCTGTAGCCCACGTCGTAGC
IL-1β	Forward	CAGGATGAGGACATGAGCACC
	Reverse	CTCTGCAGACTCAAACTCCAC
IL-6	Forward	GTACTCCAGAAGACCAGAGG
	Reverse	TGCTGGTGACAACCACGGCC
IL-8	Forward	GGCAGCCTTCCTGATTTCTG
	Reverse	CTTGGCAAAACTGCACCTTCA
MCP-1	Forward	CCAGCACCAGCACCAGCCAA
	Reverse	TGGATGCTCCAGCCGGCAAC
iNOS	Forward	CCCTTCCGAAGTTTCTGGCAGCAGC
	Reverse	GGCTGTCAGAGCCTCGTGGCTTTGG
COX2	Forward	ACGGTCCTGAACGCATTTATG
	Reverse	TTGGCCCCATTTAGCAATCTG
GAPDH	Forward	AGGTCGGTGTGAACGGATTTG
	Reverse	TGTAGACCATGTAGTTGAGGTCA

### Analysis of NO levels

NO levels were detected using the Griess reagent assay. At 24 h after the rats underwent MCAO or the microglial cells were subjected to OGD treatment, brain tissue homogenates or the microglial cell culture supernatants were mixed with equal (1:1) volumes of Griess reagent (Sigma; cat#: 23479). Absorbance was determined at 540 nm in a 96-well Spectra MAX 340PC microplate reader (Molecular Devices) and the data was analyzed using the Softmax Pro 7 software (Molecular Devices). Values were calculated using a standard curve with serial dilutions of sodium nitrite dissolved in double-distilled water.

### Measurement of ROS production

ROS levels were measured with the oxidative conversion of 2′,7′-dichlorofluorescin diacetate (DCFH-DA) into the fluorescent compound dichlorofluorescin (DCF). In brief, the microglial cell culture supernatants or brain tissue homogenates were incubated in HBSS containing 10 μM DCFH-DA (Sigma; cat#: 287810) at 37° C for 30 min. DCF levels were quantified by determining the fluorescence at excitation and emission wavelengths of 485 nm and 520 nm, respectively, on a fluorescence microplate reader (Safire2, Tecan, Switzerland).

### Measurement of iNOS activity

iNOS activity was measured by monitoring the conversion of arginine to citrulline. At 12 h after OGD treatment, an aliquot of microglial cell lysate (10 μl) was incubated with a mixture of L-[^3^H] arginine, tetrahydrobiopterin, NADPH, and flavin adenine dinucleotide. To stop the reaction, EDTA and ethylene glycol tetraacetic acid (EGTA) were sequentially added into the mixture. L-[^3^H] citrulline concentration was calculated by liquid scintillation counting. An appropriate blank containing 1 mM L-NG-nitroarginine methyl ester (L-NAME), a competitive iNOS inhibitor, was used to determine the effect from the background of the nonspecific metabolism of L-arginine. iNOS activity was calculated by the inhibitable degree of L-NAME in the EDTA-EGTA sample and expressed in units (where 1 unit = 1 pmol L-[^3^H] citrulline/mg protein/min).

### Western blot analysis

Rat brain tissues were homogenized in T-PER tissue protein extraction reagent (Pierce 78510, Rockford, IL, USA) containing protease inhibitor cocktail set III (EMD Chemicals Inc., MerckKGaA, Darmstadt, Germany). The homogenates were centrifuged at 15,000 × *g* and 4° C for 25 min and the supernatants were harvested to measure the cytosolic protein. The nuclei-rich pellets were resuspended in nuclear protein extraction buffer [10% glycerol, 1 mM dithiothreitol, 400 mM NaCl, 1 mM EDTA, 20 mM 4-(2-hydroxyethyl)-1-piperazineethanesulfonic acid (pH 7.9), and cocktail set III]. The resuspensions were centrifuged at 15,000 × *g* and 4° C for 20 min and the supernatants were harvested for nuclear protein determinations. Cytosolic and nuclear extracts of microglial cells were prepared using a nuclear extract kit (Active Motif, Carlsbad, CA, USA; cat#: 40010). The cytosolic and nuclear proteins were separated by 10% sodium dodecyl sulfate polyacrylamide gel electrophoresis and electrotransferred onto nitrocellulose membranes. After blocking with 5% nonfat milk for 1 h at room temperature, the membranes were incubated with primary antibodies overnight at 4° C followed by HRP-conjugated anti-rabbit IgG incubation for 1 h at room temperature. The protein bands were visualized by using the enhanced chemiluminescence assay kit (Pierce; cat#: 32109) and the band density was quantified by using the Quantity One software (Bio-Rad, Berkeley, CA, USA). The basal levels of the each protein were normalized by analyzing the level of β-actin or lamin B protein. The results were calculated as fold changes against to the first treatment group (fold of basal is 1 and ± SD is 0) in each experiment.

### NF-κB DNA-binding activity assay

At 24 h after the rats underwent MCAO or at 12 h after the microglial cells were subjected to OGD treatment, nuclear extracts of brain tissue samples or microglial cells were prepared using the nuclear extraction kit (Active Motif; cat#: 40010). NF-κB DNA-binding activity was analyzed by an ELISA basing on TransAM NF-κB p65 transcription factor assay kit (Active Motif; cat#: 40096) according to manufacturer’s instructions. Briefly, the NF-κB transcription factor was captured by binding to a consensus sequence 5′-GGGACTTTCC-3′ immobilized on a 96-well plate. Nuclear extract (5 μg) was added to each well and incubated with anti-NF-κB p65 antibody, followed by HRP-conjugated secondary antibody incubation. The absorbance was read on an ELISA plate scanner (CA94089, Molecular Devices) at a wavelength of 450 nm. Results were expressed as the percentage of NF-κB DNA-binding activity relative to control cells.

### Nucleosomal fragmentation assay

Cell apoptosis was quantified by assaying nucleosomal fragmentation. At 24 h after the rats underwent MCAO or the co-cultures were treated with OGD, brain tissue samples and microglia or neurons were collected to assess nucleosomal fragmentation with the Cell Death Detection ELISA PLUS kit (Sigma; cat#: 11774425001) according to the manufacturer’s protocol. To derive a nucleosomal enrichment factor, absorbance values were normalized to those of control-treated cells.

### Caspase-3 activity assay

Caspase-3 activity was detected using a Caspase-3/CPP32 colorimetric assay kit (Biovision, Palo Alto, CA, USA; cat#: K106-25) as per the manufacturer’s instructions. 1 × 10^6^ microglial cells or 1 × 10^4^ neurons (control and OGD groups) or 2 mg brain tissues (control and MCAO groups) were lysed with 50 μl of chilled lysis buffer in an ice bath for 10 min. After centrifugation at 10,000 × g for 10 min, 150 μg protein lysate (50 μl supernatant) was incubated with 50 μl of 2× reaction buffer containing 5 μl of 2 mM *N*-acetyl-Asp-Glu-Val-Asp-pNA substrate at 37° C for 2 h. Caspase-3 activity was analyzed by quantifying pNA at 405 nm with a microplate reader (Bio-Tek Instruments Inc., Winooski, VT, USA).

### Mitochondrial membrane potential analysis

The loss of mitochondrial membrane potential indicates the apoptosis, which was detected with the fluorescent cationic JC-1 dye (Beyotime, Haimen, China; cat#: C2006). The control and OGD-exposed microglial cells or neurons were stained with JC-1 in the dark for 30 min. JC-1 accumulates in the mitochondria as a monomer at low membrane potential (green fluorescence; emission at 527 nm) and as aggregates at high membrane potential (red fluorescence; emission at 590 nm). The cells were analyzed on the BD FACS Calibur flow cytometer (BD Biosciences) and the JC-1 fluorescence in different groups was determined.

### Statistical analyses

The data are expressed as mean ± standard deviation (SD) of *n* observations. Intergroup differences were determined by Student’s two-tailed unpaired *t*-test or one-way analysis of variance, followed by Dunnet’s post-hoc test when appropriate. The GraphPad statistical software (GraphPad Software, Inc., San Diego, CA, USA) was used for the analysis. P < 0.05 was considered statistically significant.

## Supplementary Material

Supplementary Figure 1
