# Supplementary material for: Subanesthetic isoflurane abates ROS-activated MAPK/NF-κB signaling to repress ischemia-induced microglia inflammation and brain injury
Source: Aging (Albany NY). 2020 Dec 28;12(24):26121–39. doi: 10.18632/aging.202349 (PMC7803578; doi:10.18632/aging.202349)
Supplement: Supplementary Figure 1 [file aging-12-202349-s001.pdf]

SUPPLEMENTARY FIGURE

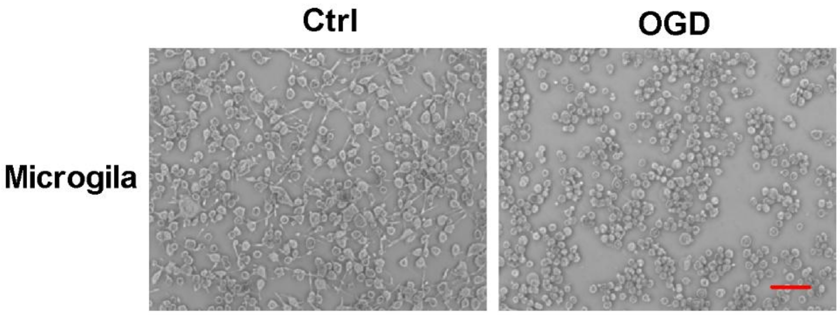

Supplementary Figure 1. The pictures of OGD-treated microglial cells in co-cultures. After co-cultures were treated with or without OGD for 3 h, the non-contact inserts (upper chamber) of transwell apparatus were removed, and the pictures of OGD-treated microglial cells (lower chamber) were taken under a microscope. Scale bar: 20 µm. Ctrl: control; OGD: oxygen and glucose deprivation.
